# Availability and Geographic Access to Hospital-Based Breast Cancer Diagnostic Services in Ghana

**DOI:** 10.1200/GO.23.00231

**Published:** 2024-02-08

**Authors:** Matthew D. Price, Anne F. Rositch, Florence Dedey, Meghan E. Mali, Kirstyn E. Brownson, Josephine Nsaful, Mamadou Tounkara, Raymond R. Price, Edward Kofi Sutherland

**Affiliations:** ^1^ Department of Epidemiology, The Johns Hopkins Bloomberg School of Public Health, Baltimore, MD; ^2^ Department of Surgery, The Johns Hopkins University School of Medicine, Baltimore, MD; ^3^ The University of Utah, Center for Global Surgery, Salt Lake City, UT; ^4^ University of Ghana Medical School, Accra, Ghana; ^5^ Department of Surgery, The University of Utah, Salt Lake City, UT; ^6^ Huntsman Cancer Institute, Salt Lake City, UT; ^7^ Intermountain Healthcare, Salt Lake City, UT; ^8^ Ensign Global College Ghana, Kpong, Ghana

## Abstract

**PURPOSE:**

Breast cancer is the most frequent cancer and second most common cause of cancer-related death in Ghana. Early detection and access to diagnostic services are vital for early treatment initiation and improved survival. This study characterizes the geographic access to hospital-based breast cancer diagnostic services in Ghana as a framework for expansion.

**METHODS:**

A cross-sectional hospital-based survey was completed in Ghana from November 2020 to October 2021. Early diagnostic services, as defined by the National Comprehensive Cancer Network (NCCN) Framework for Resource Stratification, was assessed at each hospital. Services were characterized as available >80% of the time in the previous year, <80%, or not available. ArcGIS was used to identify the proportion of the population within 20 and 45 km of services.

**RESULTS:**

Most hospitals in Ghana participated in this survey (95%; 328 of 346). Of these, 12 met full NCCN Basic criteria >80% of the time, with 43% of the population living within 45 km. Ten of the 12 met full NCCN Core criteria, and none met full NCCN Enhanced criteria. An additional 12 hospitals were identified that provide the majority of NCCN Basic services but lack select services necessary to meet this criterion. Expansion of services in these hospitals could result in an additional 20% of the population having access to NCCN Basic-level early diagnostic services within 45 km.

**CONCLUSION:**

Hospital-based services for breast cancer early diagnosis in Ghana are available but sparse. Many hospitals offer fragmented aspects of care, but only a limited number of hospitals offer the full NCCN Basic or Core level of care. Understanding current availability and geographical distribution of services provides a framework for potential targeted expansion of services.

## Teaser

An overview of breast cancer diagnostic services in Ghana provides a roadmap for targeted expansion.

## INTRODUCTION

Breast cancer is the most common cancer diagnosed worldwide and accounts for more disability-adjusted life-years lost than any other malignancy.^1-3^ Most new diagnoses of breast cancer continue to occur in high-income countries (HICs) where robust access to early detection, diagnostic, and treatment services allow for a 5-year survival that approaches 90%.^4,5^ By contrast, low- and middle-income countries lag in early detection, diagnostics, and treatment and thus carry a disproportionate share of breast cancer burden.^1,3,4,6,7^ In Ghana, these trends hold true, where breast cancer is the leading type of cancer, the second most common cause of cancer-related death, and where 5-year survival has been cited at 40%-48%.^8-12^ Most women in Ghana present with stage III or IV breast cancer; however, stage I breast cancer treated in Ghana has been found to have the same 5-year survival as in HICs.^12^

**CONTEXT**

**Key Objective**
What is the geographic availability and accessibility to early diagnostic breast cancer services at hospital facilities in Ghana, and are there regions that could benefit from targeted service expansion?
**Knowledge Generated**
Hospital-based services for breast cancer early diagnosis in Ghana were found to be available but sparse. Many hospitals offered fragmented aspects of care, but only a limited number offered the full spectrum of basic-level services outlined in the National Comprehensive Cancer Network Framework for resource-limited settings.
**Relevance**
The understanding of the availability and geographic distribution of services provides a framework for potential targeted expansion of breast cancer diagnostic services to benefit underserved populations.

In Ghana, time from symptom onset to pathologic diagnosis of breast cancer has been shown to take nearly 8 months, while delays of more than 3 months have notably been associated with later-stage presentation and worse outcomes.^13,14^ The WHO declared in their Global Breast Cancer Initiative (GBCI) 60 days from symptom onset to pathologic diagnosis as a central pillar to averting 2.5 million breast cancer deaths globally by 2040.^15,16^ To achieve this, first, a thorough understanding of the barriers to accessing breast cancer early detection and diagnostic services is required. In Ghana, several barriers have been cited, including lack of community knowledge and misconceptions of breast cancer to allow for early recognition,^17,18^ perceived and actual costs associated with seeking care,^19,20^ and distance required to travel to access available services,^21,22^ among others. Currently, geographic availability and access to breast cancer early diagnostic services in Ghana are not well defined.

Ghana is situated in West Africa with a 2021 population of 31 million people and a Human Development Index (HDI) that varies by region with areas of low development in the north (HDI, 0.539) to a region of high economic development in Greater Accra (HDI, 0.707).^23,24^ Health care in Ghana is largely centered near the two most populated cities of Accra and Kumasi, with 38% of the population residing within these cities' regions.^25^ Ghana offers universal health coverage via the National Health Insurance Scheme, although less than half of the population are enrolled.^26^

This study aims to characterize hospital-based breast cancer early diagnostic services available throughout Ghana and uses the National Comprehensive Cancer Network (NCCN) Framework for Resource Stratification as a roadmap toward expansion of these services.^7,27^ The NCCN resource-stratified guidelines provide a four-tiered framework from Basic or essential services needed to provide the basic minimal standard of care to the Full NCCN Guidelines, which incorporate enhanced and additional services that provide improvement in disease outcomes but may be cost-prohibitive in lower-resource settings.^27,28^ Our geographic analysis of breast cancer service availability identifies regions with limited access to help guide a systematic expansion of diagnostic services to close the gap from symptom onset to pathologic diagnosis of breast cancer in Ghana.

## METHODS

### Study Design

A cross sectional, in-person, hospital-based survey was conducted from November 2020 to October 2021 that evaluated the availability of breast and cervical cancer diagnostic and treatment services as defined by the NCCN framework for resource stratification.^27^ This paper reports specifically on breast cancer diagnostic services. The survey design has been previously described in detail in the study by Moustafa et al,^22^ which reports on a pilot study of this survey in the Eastern Region of the country. On the basis of the pilot study, revisions to the survey were made before the nationwide rollout.^27^

All health facilities with a hospital designation as determined by the Health Facilities Regulatory Agency (HeFRA) and the Regional Health Directorates of the Ghana Health Services (GHS) were approached for participation.^29^ Specialized hospitals such as orthopedic and psychiatric hospitals were excluded. Ten research assistants (RAs) were recruited and underwent training on how to conduct the in-person survey and were provided a general understanding of breast and cervical cancer diagnosis and care. Training was conducted virtually due to the COVID-19 pandemic. Letters were sent in advance from the Director General of the GHS through the Regional Health Directorates to all eligible hospitals describing the study purpose and indicating support from the GHS. For non-GHS hospitals, such as the private, quasi-government, and teaching hospitals, administrative approvals and support were sought from hospital management.

Missing data or inconsistent responses were reviewed with the RA, and if further clarification was indicated, the hospital was again contacted. The Eastern Region was resurveyed for this study. Institutional review board (IRB) approval was first sought from the GHS Ethics Review Committee and additionally at the IRBs of the teaching hospitals that participated in the study. The need for further IRB approval was waived by the Johns Hopkins School of Public Health IRB as the subject pertains to secondary deidentified data.

### Hospital Service Categorization

Breast cancer early diagnostic services assessed at each hospital include all those described in the NCCN Framework for Resource Stratification's Basic, Core, and Enhanced designations (Table 1). The survey distinguished services as being mostly available (>80% of the time in the previous year), somewhat available (<80%), or not at all. Descriptive statistics were generated using STATA (StataCorp, 2023, version 17, College Station, TX).

**TABLE 1:** Individual Services Required for NCCN Framework for Resource Stratification Designation

NCCN Framework for Resource Stratification^a^	Framework Goal	Minimum Required Early Diagnostic Services
Basic	Essential services needed to provide the basic minimal standard of care	Clinical breast examination Excisional OR incisional biopsy Pathologic review (in-house OR external) Estrogen receptor testing Progesterone receptor testing X-ray Diagnostic ultrasound OR diagnostic mammography
Core	Additional services that provide major improvements in disease outcome and are not cost-prohibitive	All Basic-level services Core needle biopsy OR FNA
Enhanced	Additional services that provide lesser improvements in disease outcomes and/or services that provide major improvements in disease outcomes but are cost-prohibitive	All Basic- AND Core-level services Skin punch biopsy HER2 testing CT scan Bone scan Breast MRI Genetic testing Screening AND diagnostic mammography

Abbreviations: CT, computed tomography; FNA, fine-needle aspiration; HER2, human epidermal growth factor 2; MRI, magnetic resonance imaging; NCCN, National Comprehensive Cancer Network.

^a^ The full NCCN Framework for resource stratification includes both diagnostic and treatment services; this table includes only the early diagnostic services

Using the NCCN Framework for Resource Stratification as a model, hospitals were categorized as meeting full NCCN Basic, Core, or Enhanced criteria depending on the services they provided at the time of the survey (Table 1). The full NCCN Framework includes early diagnostic and treatment services; however, only the early diagnostic services were the focus of this publication. Hospitals that met Basic, Core, or Enhanced level criteria provided all services in their respective designation (Table 1). To meet the criteria for pathologic review, the service could be provided either on-site at the health facility or with external pathology services. To have met the criteria of providing diagnostic mammography or diagnostic ultrasound, hospitals could offer either of the two modalities on-site. The NCCN Harmonized Guidelines for Sub-Saharan Africa do emphasize that where mammography is available, it should be used, but in resource-constrained settings, diagnostic ultrasound has been shown to have comparable sensitivity and specificity for invasive breast cancer.^30,31^ Genetic testing as well as all imaging services were assessed only if they were on-site.

### Geospatial Mapping and Expansion Analysis

The proportion of the population within Euclidean distances of 20 and 45 km to individual services and to the hospitals with full NCCN level services were generated in ArcGIS Pro Software.^32^ This was done in a similar format as was outlined in the study by Sanyang et al^33^ conducted in Gambia. By incorporating the 2021 WorldPop density raster, a zonal statistics tool was used to obtain the proportion of the population within the specified distances.^34^ The distances of 20 and 45 km were chosen on the basis of a South African study that identified patients who lived more than 20 km away were more likely to have advanced-stage breast cancer at diagnosis and a Ghanaian study showed that 80% of patients who had to travel more than 1 hour for care would rarely or irregularly seek care (with 45 km being the estimated distance for 1 hour of travel).^21,35^

To display the geographic impact of potential targeted expansion, all hospitals in areas with a population density of 300,000 or greater in a 45-km radius that did not have a hospital meeting NCCN Basic-level designation were examined. Hospitals in these regions that met most, but not all, NCCN Basic criteria were analyzed as potential targets for expansion of services. Further population analysis was done in ArcGIS to demonstrate the potential impact of the targeted expansion at these hospitals.

## RESULTS

Three hundred and forty-six hospitals met hospital designation criteria per HeFRA and the GHS, all of which were invited to participate. Three hundred and twenty-eight (95%) hospitals completed the survey. Each of the 16 regions had hospitals that participated in the survey, ranging from 88 in the Greater Accra Region to 3 in the Savannah Region (Table 2). Of the 18 (5% of the 346) hospitals that did not complete the survey, 15 declined to participate (Greater Accra [10], Ashanti [2], Western [2], and Eastern [1] regions), two were closed for renovation (Greater Accra Region), and one could not be located (Western North Region).

**TABLE 2:** Population of Ghana by Regions and Hospitals Surveyed by Region

Region (16)	Population (N = 31,056,871), No. (%)	Hospitals Surveyed (N = 328), No. (%)	Hospitals Not Surveyed (n = 18), No.
Ashanti	6,352,774 (21)	70 (21)	2
Greater Accra	5,318,675 (17)	88 (27)	12^a^
Eastern	3,154,986 (10)	34 (11)	1
Central	2,784,283 (9)	21 (6)	0
Western Region	2,101,432 (7)	18 (6)	2
Northern	1,999,079 (6)	13 (4)	0
Volta	1,897,386 (6)	16 (5)	0
Bono East	1,118,345 (4)	11 (3)	0
Upper East	1,116,908 (4)	11 (3)	0
Bono	1,088,238 (3)	12 (4)	0
Upper West	803,893 (3)	10 (3)	0
Western North	774,716 (2)	8 (2)	1^b^
Oti	738,791 (2)	6 (2)	0
Savannah	627,996 (2)	3 (1)	0
North East	609,894 (2)	3 (1)	0
Ahafo	569,475 (2)	4 (1)	0

^a^ Two hospitals under renovation at the time of survey administration.

^b^ One hospital that could not be located at the time of survey administration.

### Individual Services

**FIG 1:**
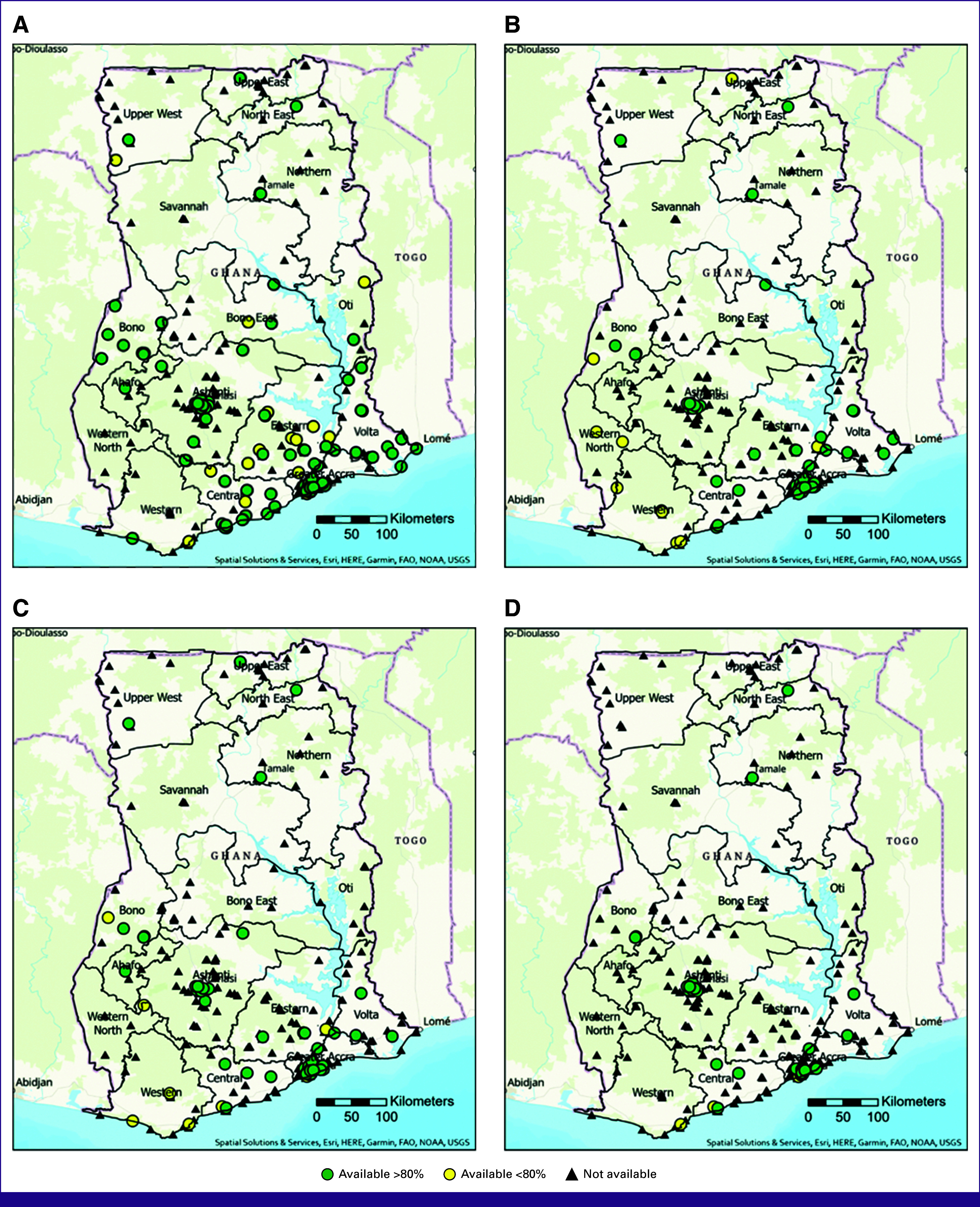
Individual biopsy service availability. (A) Excisional or incisional biopsy; (B) core needle biopsy; (C) FNA; and (D) skin punch biopsy. FAO, Food and Agriculture Organization; FNA, fine-needle aspiration; NOAA, National Oceanic and Atmospheric Administration; USGS, United States Geological Survey.

**FIG 2:**
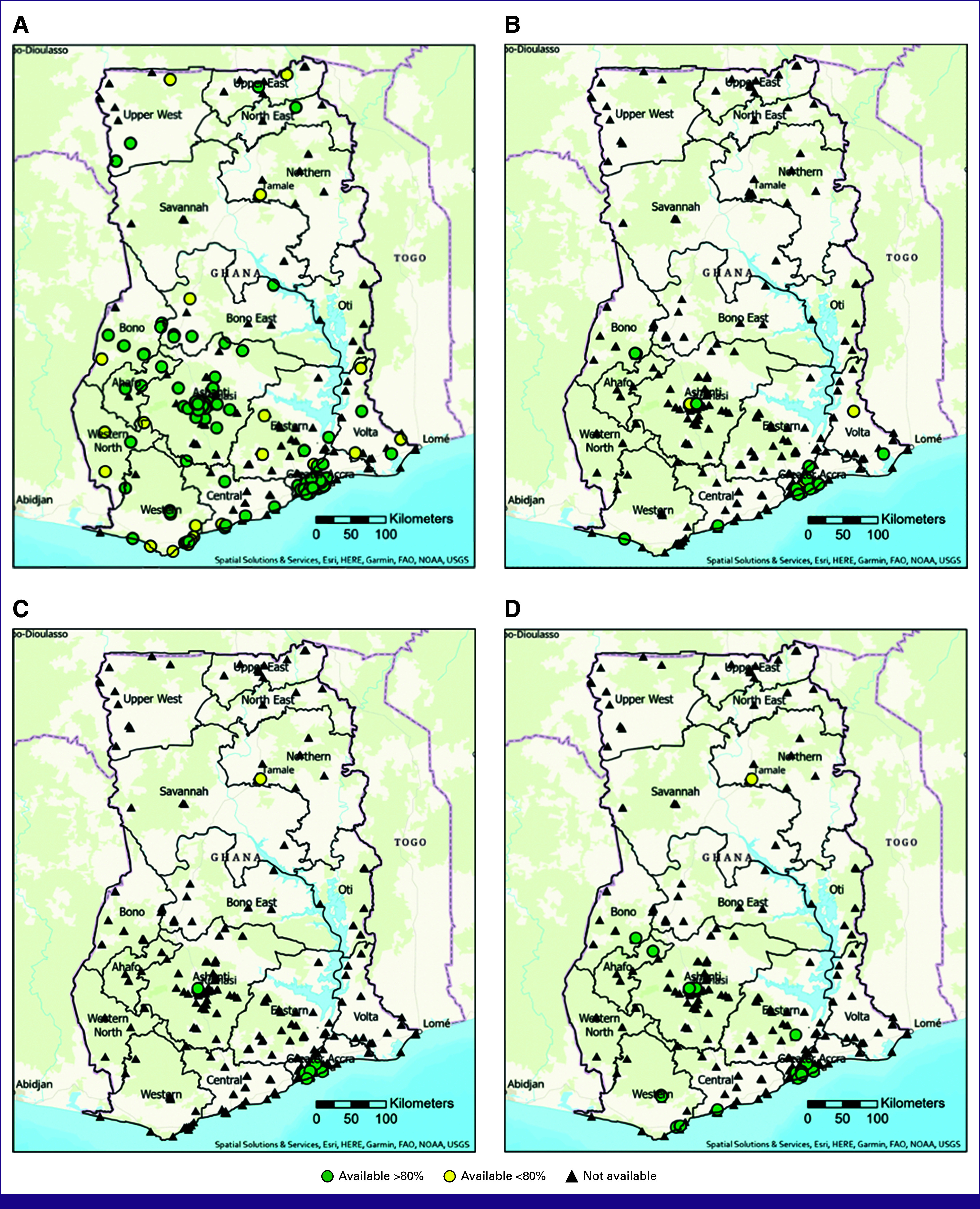
Individual imaging service availability. (A) Diagnostic breast ultrasound; (B) diagnostic mammography; (C) breast MRI; and (D) CT scan. CT, computed tomography; FAO, Food and Agriculture Organization; MRI, magnetic resonance imaging; NOAA, National Oceanic and Atmospheric Administration; USGS, United States Geological Survey.

Table 3 details the proportion of the population who live within 20 km of each individual service, both for services available greater than and less than 80% of the time. Diagnostic clinical breast examination (CBE) was available at 226 (69%) hospitals (285 if accounting for screening or diagnostic CBE). Biopsy services were less widely available, with 109 hospitals (33%) offering incisional or excisional biopsy, 64 (20%) core needle biopsy, 57 (17%) fine-needle aspiration (FNA), and 33 (10%) skin punch biopsy (Fig 1). When it came to imaging services (Fig 2), 226 (69%) hospitals reported having on-site ultrasound; however, only 132 (40%) reported usage for diagnostic breast ultrasound. Diagnostic mammography was available on-site at 21 (6.4%) hospitals. On-site computed tomography imaging was available at 19 (6%) hospitals >80% of the year, magnetic resonance imaging at seven (2%), and one hospital in Accra reported having a machine on-site for performing bone scans.

**TABLE 3:** Hospitals Reporting Individual Service Availability and Proportion of Population Within 20 km of Each Individual Service

Basic Service	No. of Hospitals Reporting Service Available >80% of the Year (proportion of population within 20 km of service), (%)	No. of Hospitals Reporting Service Available <80% of the Year (additional proportion of population within 20 km of service), (%)
Diagnostic clinical breast examination	192 (68)	34 (4)
Excisional or incisional biopsy	92 (63)	17 (7)
Pathologic review in-house or external	94 (52)	42 (10)
ER/PR testing	23 (40)	29 (10)
X-ray	156 (75)	16 (2)
Diagnostic ultrasound (breast)	104 (56)	28 (8)
Diagnostic mammography	17 (35)	4 (2)
Core services		
Core needle biopsy	53 (43)	11 (6)
Fine-needle aspiration	48 (45)	9 (5)
Enhanced services		
Skin punch biopsy	30 (38)	3 (4)
HER2 testing	27 (40)	18 (4)
CT scan	19 (37)	4 (2)
Bone scan	1 (13)	NA
Breast MRI	6 (29)	1 (1)
Genetic testing (onsite)	NA	NA

Abbreviations: CT, computed tomography; ER, estrogen receptor; HER2, human epidermal growth factor 2; MRI, magnetic resonance imaging; NA, not available; PR, progesterone receptor.

**FIG 3:**
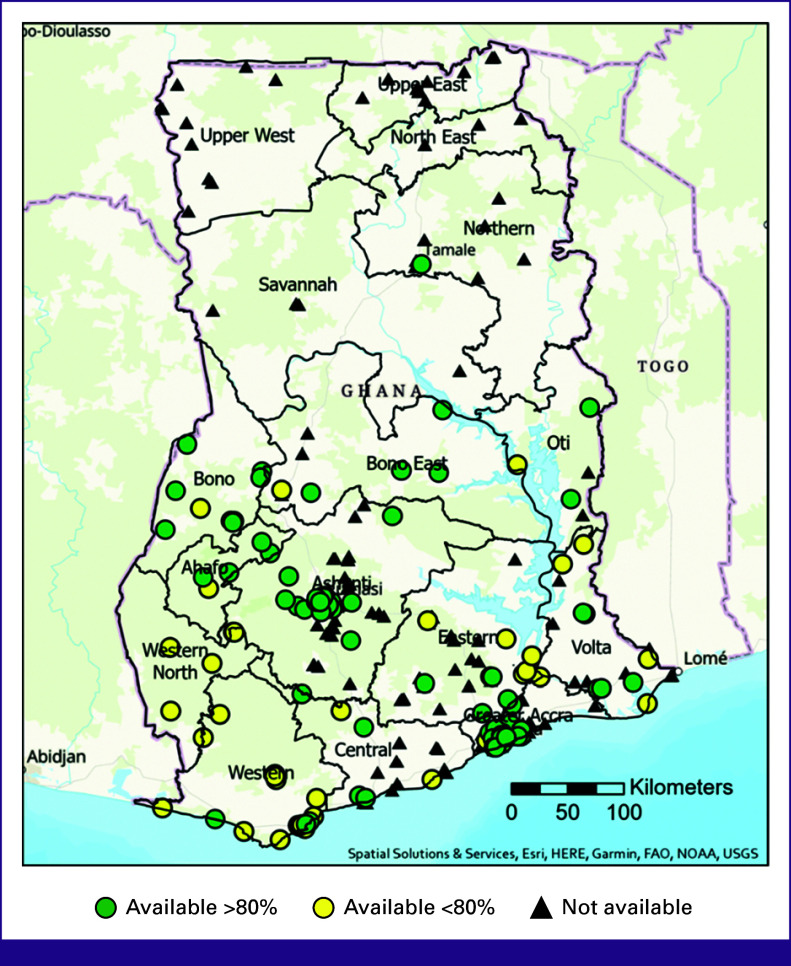
Pathology service availability. FAO, Food and Agriculture Organization; NOAA, National Oceanic and Atmospheric Administration; USGS, United States Geological Survey.

**FIG 4:**
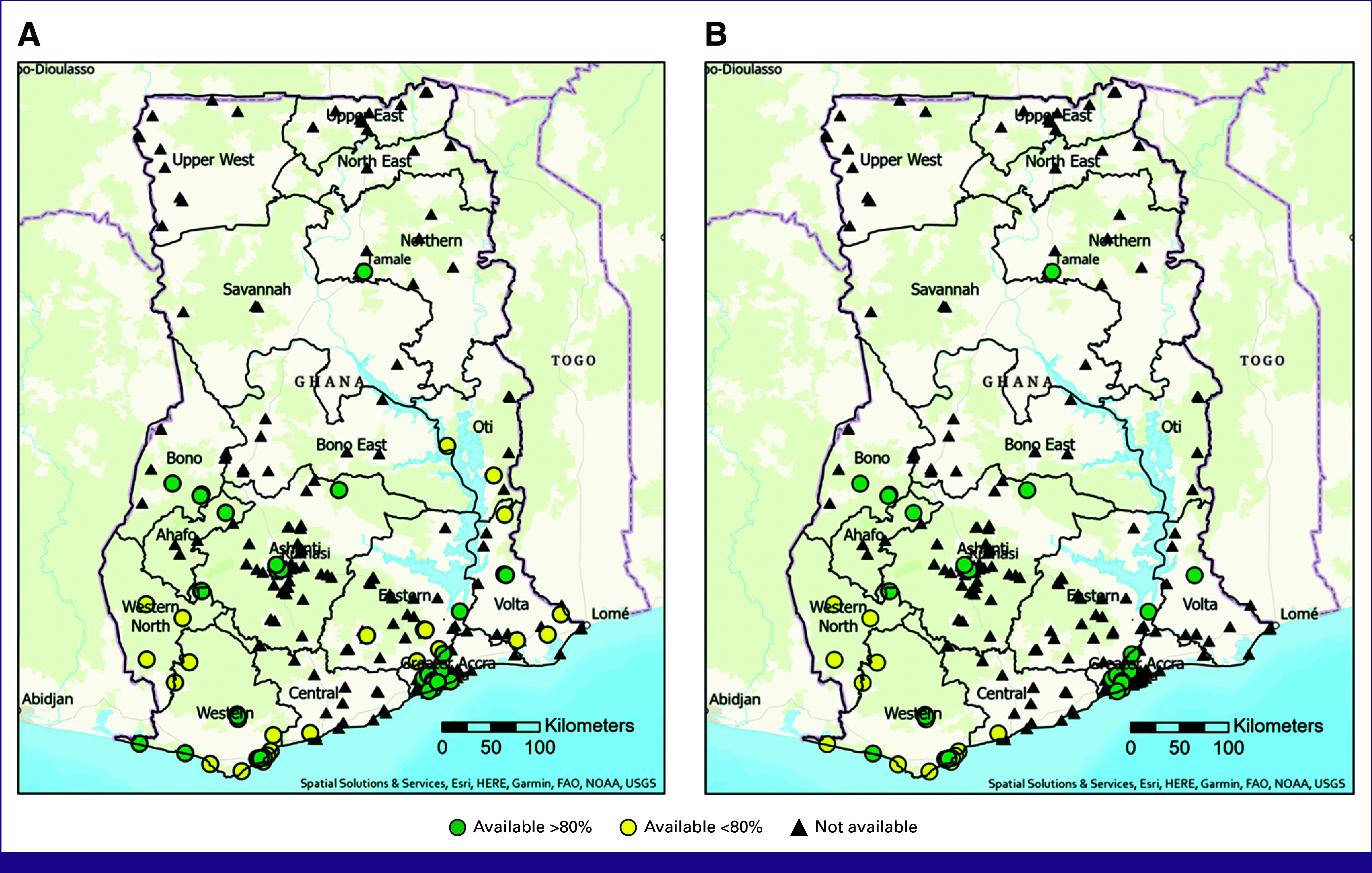
Availability of breast cancer receptor testing. (A) Estrogen or progesterone receptor testing and (B) HER2 testing (IHC or FISH). FAO, Food and Agriculture Organization; FISH, fluorescence in situ hybridization; HER2, human epidermal growth factor 2; IHC, immunohistochemistry; NOAA, National Oceanic and Atmospheric Administration; USGS, United States Geological Survey.

Internal pathologic review was available at nine (3%) hospitals, with another 127 (39%) hospitals reporting access to external pathology services. Pathology services were available at 94 (29%) hospitals >80% of the year (Fig 3). There were two main hospitals that functioned as multiregional receiving centers with on-site pathologic services, with 65 hospitals sending their pathology specimens to a central hospital in Kumasi and 30 hospitals sending their specimens to a single hospital in Accra. These two hospitals account for 70% of the breast cancer pathologic services throughout the country. Estrogen receptor (ER) or progesterone receptor (PR) hormone testing was offered at 23 (7%) hospitals >80% of the time (Fig 4). Human epidermal growth factor 2 testing with immunohistochemistry and fluorescence in situ hybridization was offered at 19 (6%) hospitals >80% of the year. No hospitals reported access to on-site genetic testing.

### NCCN Resource Stratification

**FIG 5:**
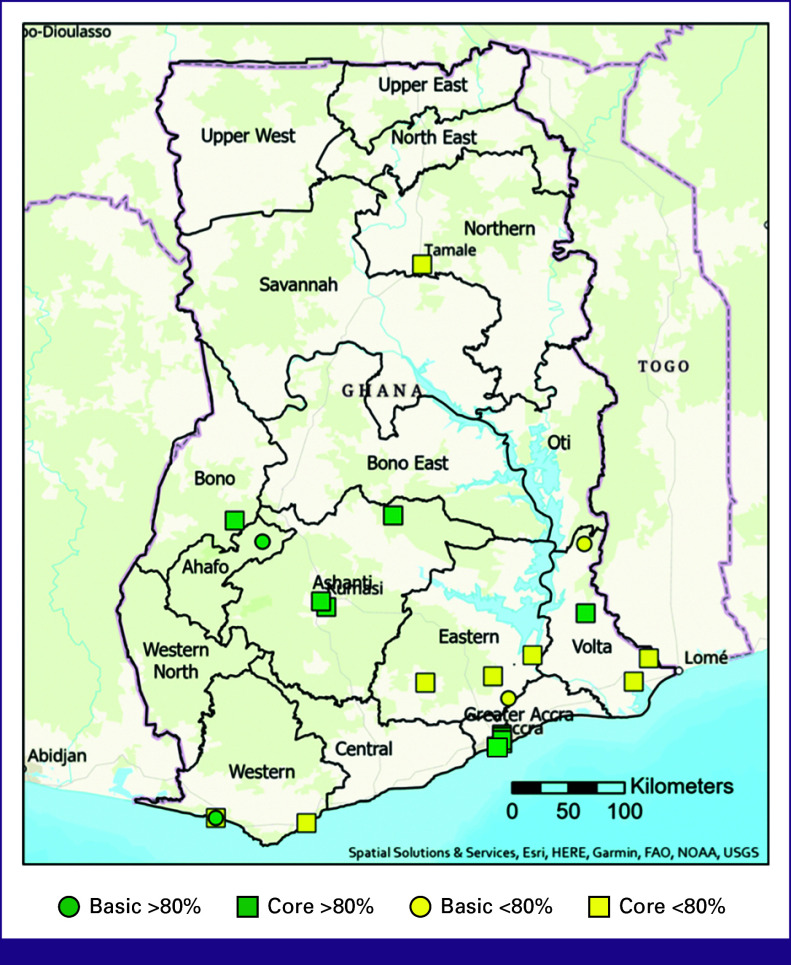
Complete NCCN level service availability. Green indicating all NCCN level services are available more than 80% of the year; yellow indicating all services available, but one or several are available <80% of the year. FAO, Food and Agriculture Organization; NOAA, National Oceanic and Atmospheric Administration; NCCN, National Comprehensive Cancer Network; USGS, United States Geological Survey.

Of the 328 hospitals, 13 (4%) met NCCN Basic criteria and offered all required early diagnostic services >80% of the time in the year preceding the survey, with 30% of the population living within 20 km (Fig 5) of one of these hospitals. Of these 13, 11 (3%) met NCCN Core criteria by also providing FNA or core needle biopsy. If including hospitals with one or more of the required services being available <80%, this would increase to 23 (7%) hospitals meeting NCCN Basic criteria, of which 20 (6%) would meet NCCN Core criteria.

No hospital met NCCN Enhanced criteria based on the NCCN Guidelines for Resource Stratification. Two hospitals, one in Kumasi and one in Accra, would have met all Enhanced-level criteria >80% of the time if they performed bone scans and genetic testing. A second hospital in Accra met the majority of the NCCN Enhanced criteria >80% of the year, including having an on-site bone scan device, but reported skin punch biopsy being available <80% of the year because of the kits not always being available, and also did not report offering genetic testing (on-site).

### Expansion Analysis

**FIG 6:**
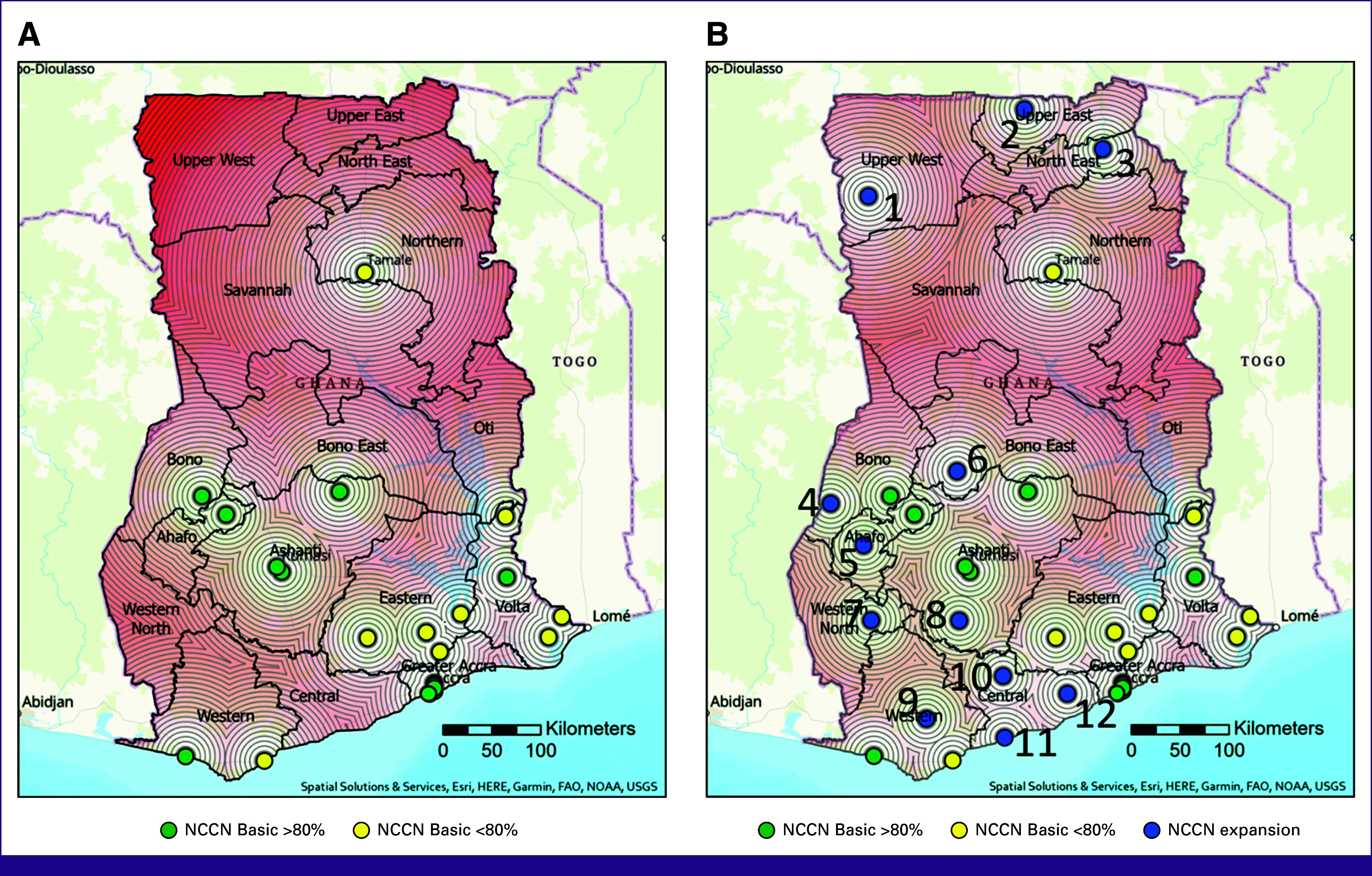
(A) Availability of NCCN Basic breast cancer early diagnostic services with 5-km concentric ring buffers with progressively darker shades of red highlighting regions further from care. (B) Current NCCN Basic service availability with expansion hospitals in blue with number labels to correlate with Table 4. FAO, Food and Agriculture Organization; NOAA, National Oceanic and Atmospheric Administration; NCCN, National Comprehensive Cancer Network; USGS, United States Geological Survey.

In our analysis of population-dense regions, 12 hospitals were identified that were lacking in only a few services to meet NCCN Basic criteria. Hospitals in the northern regions lacked access to pathology services and ER and PR testing. Similar challenges were identified in the Western North, Bono, and Central regions with pathology services available <80% of the time at all hospitals and no excisional or incisional biopsy was reported as available. Of these 12 hospitals, several lacked diagnostic breast ultrasound but did have an abdominal ultrasound available on-site. Hypothetical expansion of specific services to these 12 hospitals would result in an additional 20% of the population having access to a hospital with full NCCN Basic-level breast cancer early diagnostic services within 45 km (Fig 6; Table 4).

**TABLE 4:** Services Needed to Meet Full-Resource-Stratified NCCN Basic Criteria for Early Diagnosis at Hospitals in Densely Populated Areas; Correlate With Figure 6B

Hospital No.	Services Available	Services Needed to Meet NCCN Basic Criteria	Population Within 45 km
1	CBE, excisional biopsy, X-ray	Pathology, ER/PR testing, breast ultrasound^a^	325,207
2	CBE, excisional biopsy, X-ray	Pathology, ER/PR testing, breast ultrasound^a^	592,890
3	CBE, breast ultrasound, excisional biopsy, incisional biopsy, X-ray	Pathology, ER/PR testing	505,502
4	CBE, pathology, excisional/incisional biopsy, X-ray	ER/PR testing, breast ultrasound^a^	341,105
5	CBE, breast ultrasound, pathology, excisional biopsy	ER/PR testing, X-ray	406,183
6	CBE, breast ultrasound, X-ray, pathology	ER/PR testing, incisional/excisional biopsy	461,060
7	CBE, breast ultrasound <80, ER/PR testing, pathology <80%, X-ray	Incisional/excisional biopsy	616,926
8	CBE, excisional/incisional biopsy, X-ray	Pathology, ER/PR testing, breast ultrasound^a^	596,382
9	CBE, breast ultrasound <80, pathology <80, ER/PR testing, X-ray	Incisional/excisional biopsy	291,233
10	CBE, breast ultrasound, pathology, excisional/incisional biopsy, X-ray	ER/PR testing	729,714
11	CBE, breast ultrasound, excisional/incisional biopsy, X-ray	ER/PR testing	868,458
12	CBE, excisional/incisional biopsy, X-ray	Pathology, ER/PR testing, breast ultrasound	837,877

Abbreviations: CBE, clinical breast examination; ER/PR testing, estrogen and progesterone testing; NCCN, National Comprehensive Cancer Network.

^a^ Abdominal ultrasound was reported as available >80% of the time.

## DISCUSSION

Timely access to effective early diagnostic services is an essential component to achieving long-term survival for those with breast cancer.^15,16,36^ As breast cancer is now the leading type of cancer diagnosed globally, ensuring equitable access to these services has become even more critical.^1,3^ Achieving equitable access requires a thorough understanding of the current services available. In this study, we demonstrate that many early diagnostic services are available throughout Ghana, but few hospitals offer all NCCN Basic-level services. Fragmentation of early diagnostic services can be frustrating to navigate and lead to delays in diagnosis.^36,37^ We also demonstrate that through mapping of available services, and by using the NCCN's Framework for Resource Stratification, hospitals can be identified for targeted resource expansion to become regional diagnostic centers and thereby simplify and consolidate steps of care for patients.

Our study identified that a woman with a concerning breast mass in Ghana can undergo a CBE in a hospital in nearly every region. Additionally, 64% of the population have access to diagnostic breast ultrasound within 20 km. However, biopsy services vary in geographic access, and availability was largely concentrated in the southern regions. Only a single hospital in the northern half of the country reported pathology service availability. Access to the full spectrum of Basic-level early diagnostic services within a single hospital was only available within 20 km for 30% of the population. This stepwise evaluation of having to seek care from multiple different hospitals can make it challenging to achieve the WHO GBCI's standard of 60 days from symptom onset to pathologic diagnosis and highlights the need to have geographically centralized centers for breast cancer care, or at least regional pathology hubs.^15^

The purpose of this study is not to highlight where services are lacking but rather to highlight areas with existing partial services in geographically populated areas that could serve as locations for targeted service expansion. A significant challenge in expanding access to breast cancer services is the high cost of diagnostic equipment and the low number of specialists.^38^ For example, most northern regions of Ghana lack access to pathology and ER/PR testing despite having the capability to perform biopsies. Establishing reliable external pathology referral pathways to three hospitals could allow for another 1.4 million individuals having all Basic-level early diagnostic services present within 45 km. Similarly, the expansion of select services to nine hospitals in the Central, Western North, and Bono regions could bring centralized early diagnostic services to another 5.2 million individuals. Increasing geographically accessible diagnostic centers has been highlighted as a necessary component to facilitate timely diagnoses and avert 2.5 million breast cancer deaths by 2040.^15^

Hospital-based diagnostic mammography in Ghana is concentrated in the southern third of the country, with a total of 21 mammography machines spanning from Accra to Kumasi. Diagnostic mammography is distinct from screening mammography, which is done to identify asymptomatic disease. Many HICs have achieved great success in improving breast cancer survival with national mammography screening campaigns. However, a phased approach of first building up early diagnostic capacity, particularly in areas where most women present with late-stage disease, is seen as an initial step before implementing population-based mammography screening.^36,39,40^

There are many barriers beyond the geographical aspect that deserve further mention. Notably, the relationship between development, income disparities among regions, and their potential impact on access conditions deserves attention. We recognize the importance of considering social determinants of health, such as age, socioeconomic and educative levels, dependency ratios, and cultural misconceptions, which can profoundly influence women's access to timely diagnosis. To address this, a transdisciplinary approach should be emphasized when proposing interventions beyond spatial criteria. Such an approach will prevent underutilization of investments by considering the broader context of health care accessibility. Furthermore, only 4% of facilities met NCCN Basic criteria, pointing to a presumed underdiagnosis of breast cancer in the country and raises questions about the optimal utilization of resources when planning service expansion. Exploring these implications in more detail is critical to achieving equitable access to early diagnostic services.

A limitation of this study is that the survey focused solely on hospital-based services. Early diagnostic services provided by surrounding smaller health care facilities (eg, clinics) were not assessed. It is worth noting, however, that these smaller health care facilities are generally less equipped than hospitals, which are of a higher resource status per HeFRA designation requirements. Additionally, imaging services were only assessed if available on-site at the hospital. Privately owned imaging modalities could be available to patients off-site and in proximity to the hospitals. Further qualitative analysis may also shed light on intrinsic challenges hospitals face, for example, since the survey, one hospital has noted that although they reported not having incisional or excisional biopsy, it was not due to lack of capability, but due to referring patients to another facility where core needle biopsy could be obtained. Although the survey results were considered accurate at the time they were reported, it is important to acknowledge that there may have been changes in individual hospital services since completion of the survey that are not reflected in these results. Finally, this study only focuses on the early diagnostic portion compiled by the survey. Expansion of diagnostic services should always occur with concomitant expansion of treatment services. Analysis and presentation of treatment services are currently being performed by additional members of the project team.

In conclusion, this study provides a comprehensive assessment of breast cancer hospital-based early diagnostic services throughout Ghana and provides a roadmap for potential targeted expansion of these services to deliver quality care consistent with NCCN Framework Guidelines. Expansion of early diagnostic services should be done in tandem with expansion of treatment services and with public education and awareness campaigns.^36,37,40^ Establishing centralized centers with the full spectrum of breast cancer services can streamline patient care and lead to earlier pathologic diagnosis and treatment initiation, ultimately resulting in better cancer survival outcomes.
